# Retraction Note: Meeting Abstract: A post-hoc qualitative analysis of real time heads-up pollen counting versus traditional microscopy counting in the environmental exposure unit (EEU)

**DOI:** 10.1186/s13223-015-0079-8

**Published:** 2015-04-17

**Authors:** Lisa M Steacy, Terry JB Walker, Barnaby Hobsbawn, Jenny Thiele, Anne K Ellis

**Affiliations:** Allergy Research Unit, Kingston General Hospital, Ontario, Canada; Department of Medicine, Queen’s University, Kingston, Ontario Canada

## Retraction

This meeting abstract [1], published in the Canadian Society of Allergy and Clinical Immunology and AllerGen Abstracts 2014 supplement, has been retracted from *Allergy, Asthma & Clinical Immunology* by the authors. The meeting abstract has previously been published in *Journal of Allergy and Clinical Immunology* [2] and was inadvertently submitted to *Allergy, Asthma & Clinical Immunology* as part of the supplement. The authors apologise for any inconvenience that this may have caused.
